# The toxicity assessment of extract of *Peganum harmala* L. seeds in *Caenorhabditis elegans*

**DOI:** 10.1186/s12906-020-03051-x

**Published:** 2020-08-17

**Authors:** Xiangzhen Miao, Xiao Zhang, Yanyan Yuan, Yali Zhang, Jian Gao, Nianxin Kang, Xinkui Liu, Jiarui Wu, Yonggang Liu, Peng Tan

**Affiliations:** School of Chinese Pharmacy, Beijing University of Chinese Medicine, No. 11, Bei San Huan Dong Lu, Chaoyang District, Beijing, 100029 China

**Keywords:** *Peganum harmala* L., *Caenorhabditis elegans*, Toxicity

## Abstract

**Background:**

*Peganum harmala* L. is a medicinal herb extensively used in traditional Chinese medicine (TCM). So far, relevant reports on the toxicity of *Peganum harmala* L. seeds (PHS) are hardly available. Especially, we still know little about the in vivo mechanism for PHS toxicity. This study aims to evaluate the toxicity effects of PHS in *Caenorhabditis elegans* (*C. elegans*), investigate the possible mechanism of the toxicity effects of PHS, and provide reference for the pharmacological research of PHS.

**Methods:**

In the present study, the *C. elegans* was exposed to 0.25, 0.50, 1.00 mg/mL of PHS in nematode growth medium (NGM) at 22 °C in the presence of food. Lethality, lifespan, growth, reproduction, and locomotion behavior assays were performed to evaluate the toxicity effects of PHS in *C. elegans*. We then determined the mechanism of the toxicity effect of PHS by quantitative real-time polymerase chain reaction (qRT-PCR), acetylcholinesterase (AChE) activity assay, and oxidative stress resistance assays. The main components of PHS were detected by high performance liquid chromatography (HPLC).

**Results:**

Compared with the control group, the lethality of *C. elegans* was significantly increased when they were exposed to the ethanol extract of PHS at 0.25, 0.50 and 1.00 mg/mL (*P* < 0.01), and the mean lifespan was significantly decreased (*P* < 0.01). We also observed that PHS exposure could induce the toxicity on body length, brood size, and locomotion behavior.

**Conclusion:**

Our study shows that the ethanol extract of PHS exerts obvious toxic effects on *C. elegans*, which would provide new ideas and methods for the biological evaluation of the toxicity of Chinese medicinal materials.

## Background

*Peganum harmala* L. is widely distributed in the Central Asia, North Africa and Middle East [1]. It is a common medicinal material in traditional Chinese medicine for the treatment of cough, diabetes, depression, hypertension, jaundice, Parkinson’s and Alzheimer’s diseases, and many other human ailments. It has a variety of biological activities, including anti-cancer [2], anti-inflammatory, antibacterial [3], anti-depression [4], antioxidant [5],cholinesterase and monoamine oxidase inhibitory activities, antitumor, anti-hypertension, anticoagulant, antidiabetic, antimicrobial, insecticidal, antiparasidal, anti-leishmaniasis [6].

PHS has long been known to possess hypothermic and remarkably hallucinogenic properties [7]. The main chemical constituents of PHS are alkaloids, flavonoids and anthraquinones. Harmaline and harmine are the two main alkaloidal constituents in PHS [8], which can accumulate in dry seeds at 4.3 and 5.6% (w/w). Harmaline and harmine are primary toxic alkaloids in the PHS. The toxicity of harmaline is almost twice than harmine and in moderate doses causes tremors and clonic convulsions, but with no increase in spinal reflex excitability [9]. Peganumine B showed potential inhibitory activity against both AChE and BChE respectively. Peganumine C, peganumine D, and peganumine I were found to have selective inhibitory activity against AChE respectively. Peganumine G and peganumine H showed significant cytotoxicity against a ZR-75-1 cell line respectively [10]. Beta-carboline alkaloids may result in toxic effects, namely visual and auditory hallucinations, locomotor ataxia, nausea, vomiting, confusion and agitation [11]. So far, relevant reports on the toxicity of PHS are hardly available. At present, there are few studies on the toxic mechanism of PHS. Therefore, the present study aims to evaluate the toxicity effects of PHS by *C. elegans.* Harmine could ameliorate impaired memory by enhancement of cholinergic neurotransmission via inhibiting the activity of acetylcholinesterase [12]. Harmaline and harmine could effectively ameliorate memory deficits in scopolamine-induced mice, both of them exhibited an enhancement in cholinergic function by inhibiting AChE [13].

*C. elegans* is a common nematode living in the soil. *C. elegans* can provide a useful toxicity test system that is best suitable for interrogating in vivo problems. *C. elegans* has been used in many biological researches, such as genomics, cell biology, neuroscience and aging. The *C. elegans* is characterized by short life cycle, compact genome, stereotypical development, ease of propagation and small size [14], which serves as a remarkable advantage in the toxicity study of TCM.

In this study, the *C. elegans* system was used to evaluate the toxicity of PHS ethanol extract in vivo. In addition, we also explored the possible mechanism of PHS toxicity to *C. elegans*. Our data here will help understand the possible mechanisms of PHS toxicity and in turn confirms that the *C. elegans* is an effectual model when used for assessing adverse effects of Chinese medicinal materials.

## Methods

### Plant materials

The PHS (Voucher specimen number:14032101) were purchased from Shenzhou Pharmacy and then authenticated by Hongliang Zhang, Xinjiang medical university. The content of harmaline was 96.76 mg/g and the content of harmine was 98.05 mg/g. The reference material has been deposited in the School of Chinese Pharmacy, Beijing University of Chinese Medicine.

Alkaloids are the main chemical components in PHS, alkaloids could be extracted by the ethanol fraction. After crushing the raw products of PHS, adding 10 times of 95% ethanol ultrasound (540 W, 40 kHz) ultrasonic extraction twice, each time for 1 h, the filtrate was dried after filtration, and the ethanol extract of the raw products of PHS was obtained and preserved at 4 °C. Deionized water is used to mix the required concentration.

### Chemicals and reagents

Agar powder, tryptone, 1,1-diphenyl-2-trinitrophenylhydrazine (DPPH), glutathione and 2′,7′-Dichlorofluorescein were purchased from Solarbio Co. (Beijing, China). Cholesterol, paraquat and Vitamin C (Vc) were purchased from Sigma-Aldrich (St. Louis, MO). Methanol was purchased from Merck KGaA (Germany). Biowest Agarose was purchased from wobisen technology co. (Beijing, China). All the other chemicals were standard commercial products of analytical-reagent grade.

### Strain preparation and culture methods

We used wild-type N2 (Bristol) in this study, obtained from the Institute of Genetics and Developmental Biology. *C. elegans* were stored in the NGM, which was cultured at 22 °C and seeded with *Escherichia coli* OP50 (*E. coli* OP50) [15]. Under aseptic condition, 100 spawning *C. elegans* were selected on a new culture medium covered with *E.coli* OP50, and all the *C. elegans* were picked out after laying eggs for 2 h. The spawning plate was cultured in an aseptic biochemical incubator (LRH-250, Shanghai China) at 22 °C for a specific period [16].

### Lethality and lifespan assays

We chose 0.25, 0.50, 1.00 mg/mL as the exposure concentration of PHS. These specific drug concentrations were based on previous experimental research, Tan Lab, unpublished data. Thirty *C. elegans* at L4 stage were added to NGM of PHS ethanol extract of 0.5, 1.0 and 2.5 mg/mL, and cultured at 22 °C. The number of deaths of *C. elegans* was observed and recorded every 24 h. In order to eliminate the mixture of the tested *C. elegans* and its newly produced larvae, the live *C. elegans* were picked out every 24 h on a new culture plate containing the same drug concentration until all the *C. elegans* died. The mortality standard of *C. elegans* is that the worm itself is incapacitated and does not react to touching many times. Three groups of parallel tests were performed for each drug concentration.

Thirty *C. elegans* were cultured on normal NGM after 24 h exposure to ethanol extract from different concentrations of PHS. The number of deaths of *C. elegans* was observed and recorded every 24 h. When the survival number of *C. elegans* was 15, the test days were recorded, that is the Mean lifespan.

### Growth and reproduction assays

According to previous literature [17], the *C. elegans* was treated at 50 °C for 10 min after exposure to ethanol extract from PHS of different concentrations for 24 h, observed and photographed under stereoscopic microscope (Nikon, Japan), and measured the length of *C. elegans* by Image J software (Bethesda, MD, USA). Thirty *C. elegans* were measured in each concentration group.

Thirty *C. elegans* exposed to different concentrations of PHS ethanol extract for 24 h were selected into the normal NGM, one in each medium, and transferred the medium every 12 h until the end of the spawning period of *C. elegans* [18]. Record the number of all offspring that it hatches as an indicator of the number of offspring.

### Locomotion behavior assay

Evaluation of locomotion behavior through endpoints of head thrash and body bend [16]. Added 60 μL M9 to the medium which without *E. coli* OP50. Picked thirty *C. elegans* exposed to different concentrations of PHS ethanol extract for 24 h into the NGM. After 1 min recovery, the number of head thrash of *C. elegans* in 1 min was recorded. Head thrash of *C. elegans* was defined swing from one side to the other and then back.

Thirty *C. elegans* exposed to different concentrations of PHS ethanol extract for 24 h were placed on the NGM without *E.coli* OP50, and their body bend times within 20 s were counted. Body bend was counted as a change in the direction of the part of *C. elegans* corresponding to the posterior bulb of the pharynx along the y-axis, assuming that nematode was traveling along the x-axis.

### AChE activity assay

The *C. elegans* were collected into a 15 mL centrifuge tube. Centrifuged at 500 rpm for 15 min. Discarded the supernatant and wash 3 times with M9 buffer. Rinsed once with double distilled water, then transfer into a 1.5 mL centrifuge tube. Centrifuged at 3000 rpm for 10 min, discard the supernatant, stored at − 80 °C for determination of AChE activity. AChE activity was measured by the Ellman method [19]: at m(*C. elegans*): V (PBS buffer) ratio of 1: 5, added pre-chilled PBS buffer (0.1 mol/L, pH 7. 4), homogenized in a glass homogenizer under low temperature environment. Centrifuged at 4 °C,12000 rpm for 20 min, then took the supernatant for testing. The Bradford method [20] was used to determine the protein quality. Bovine serum albumin as a standard to calculate the specific activity of AChE.

### Heat stress resistance assay

The *C. elegans* synchronized in the L4 stage was placed in NGM containing drugs of different mass concentrations, 3 NGM per group, 10 *C. elegans* per NGM, and control group was set up. In the drug-administered group, *C. elegans* was treated with drug for 24 h and placed in a 35 °C incubator at the same time as the control group. The death number and survival number of *C. elegans* were counted every 12 h until all the *C. elegans* died. The death standard is described above [21]. Survival graphs were according to literature [22].

### Oxidative stress resistance assay

The *C. elegans* synchronized in the L4 stage was placed in NGM containing different concentrations of drugs, 3 NGM per group, 10 *C. elegans* per NGM, and control group was set up. We measured *C. elegans* survival when exposed to paraquat. After 24 h of development, the *C. elegans* were transferred to the NGM containing 50 mM paraquat, and the survival number of *C. elegans* was recorded every 2 h until all the *C. elegans* died. The death standard is described above [16].

### qRT-PCR assay

We used TRIzol®Reagent (Thermo Fisher Scientific) to extract total *C. elegans* RNA. Using total RNA as template, cDNA was synthesized by invitrogen reverse transcription kit (Thermo Fisher Scientific). The process of fluorescence quantitative PCR includes design and synthesis of primers, fluorescence quantitative PCR, PCR product agarose electrophoresis (Bio-rad Sub-Cell®Model 192 Cell,USA), preparation of melting curve (ABI PRISM 7500, USA). qRT-PCR was used to determine the relative quantification of the targeted genes in comparison to the reference *GAPDH* gene, and the results were expressed as the relative expression ratio (between targeted gene and internal control *GAPDH*). The designed primers for targeted genes and reference *GAPDH* gene were shown as follows (Table 1).

**Table 1:** Primers used for qRT-PCR

Gene	Forward primer	Reverse primer
*GAPDH*	CGTCAAGCTCGTCTCTTGGT	AGAGGTCACTTCAAGCTCTTTTCT
*tub-1*	GTCATCAAAGTGCGGGAAGC	ACTGCATACGTGGTTGAGCA
*ctl-2*	ACACTCATTTCCACCGCCTT	TCCCAGAATTGACGGGGTTG
*sod-3*	CCACCTGTGCAAACCAGGAT	CATGGACATAGTCTGGGCGG
*hsp-16.1*	GTCTCGCAGTTCAAGCCAGA	TCGCTTCCTTCTTTGGTGCT
*hsp-16.2*	TACCACTATTTCCGTCCAGCTCA	TGTTCTCCTTGGATTGATAGCGTA

### DPPH-scavenging capacity assay

Using Vc as a control, the antioxidant activity of PHS was studied by DPPH method, and their half-inhibitory concentration values (IC_50_) were calculated. The absorbance of PHS at 517 nm was measured by UV–visible spectrophotometer (Hitachi, Japan) [23]. The percentage of free radical scavenging activity is determined by the difference in absorbance of DPPH between the control and the sample.

### Analysis of the main chemical components in raw-processed PHS

HPLC system (Waters, USA) was used to analyze the chemical composition of PHS. The separation was carried out on an Agilent SB-C_18_ column (4.6 mm × 150 mm, 5 μm) and the mobile phase consisted of methanol (A) and 0.5% ammonium acetate (B). The isocratic elution conditions were 47% (A) and the column temperature was set at 25 °C with a flow rate of 1.0 mL/min. The detection wavelength was set to 325 nm. The injection volume was 10 μL. Comparing the HPLC retention time and all target peaks to the peak hold time of the standard, we determined the major components of the prepared PHS.

### Statistical analysis

All experiments were repeated 3 times in parallel, and the data were expressed as mean ± standard deviation. Data were analyzed by SPSS 16.0 software (SPSS Inc., Chicago, USA) and GraphPad Prism 5 (Prism, GraphPad Software, San Diego, CA). Differences between groups were analyzed by *t* test. *P* < 0.05 was considered to be statistically significant.

## Results

### Effects of PHS exposure on lethality and lifespan of *C. elegans*

**Fig. 1:**
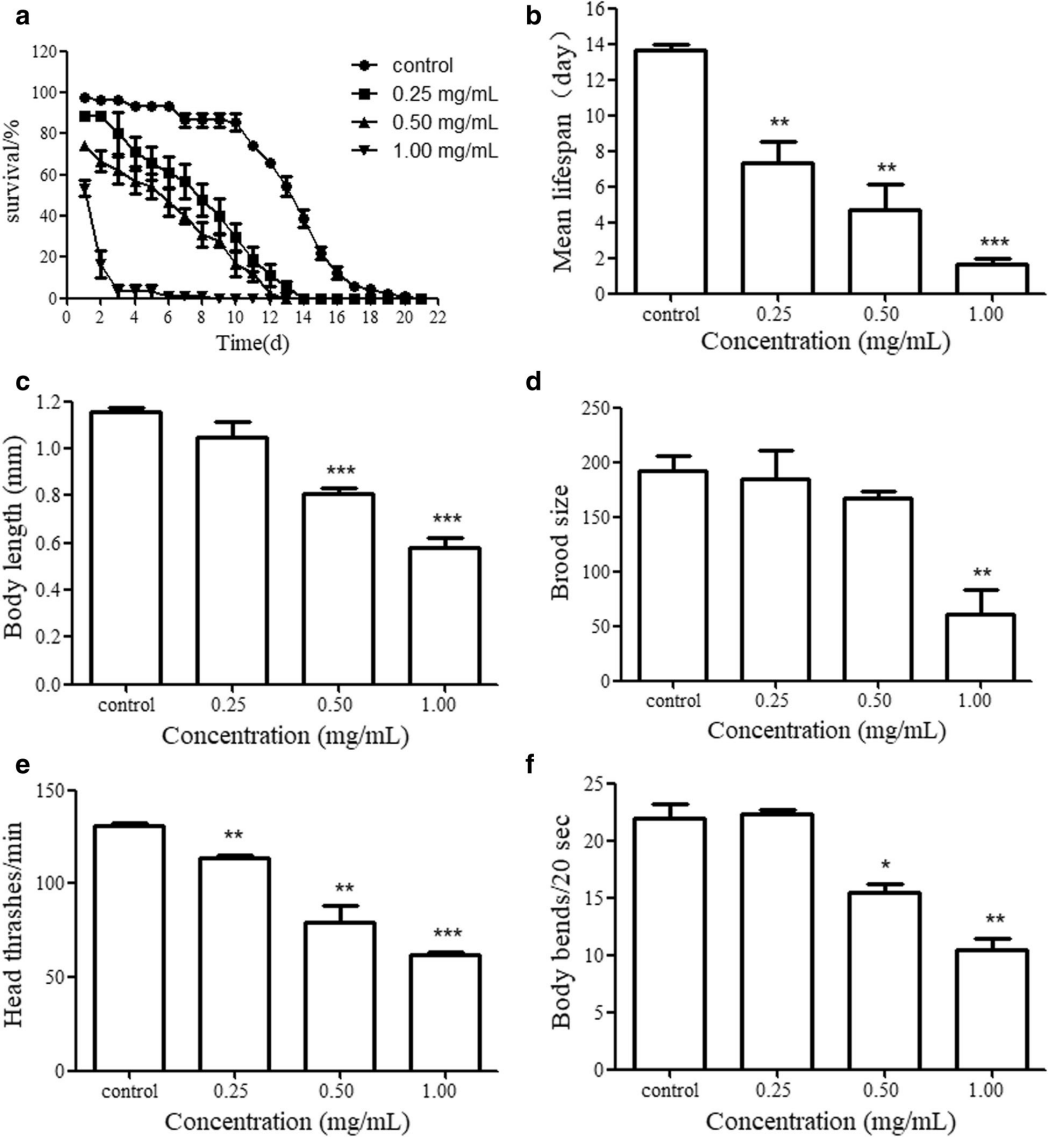
**a** Effects of PHS exposure on lethality **b** Effects of PHS exposure on lifespan **c** Effects of PHS exposure on body length **d** Effects of PHS exposure on brood size **e** Effects of PHS exposure on head thrash **f** Effects of PHS exposure on body bend Exposures were performed from L4-larvae to young adult. PHS, seeds of *Peganum harmala* L.. *C.elegans* L4-larvae to young adults were exposed to PHS. *P < 0.05, **P < 0.01, ***P < 0.001. The “control” is normal N2 worms

The analysis method of lethality of *C. elegans* is simple, and the experimental period is short and effective. Lethality is the basis for the analysis of toxicological toxicity using *C. elegans.* Lifespan is an important indicator of the long-term effects of specific toxicants. We assessed prolonged exposure to PHS in *C. elegans* L4 stage to young adults. After prolonged exposure to the examined concentrations of PHS, compared with the control group, the lethality of *C. elegans* was significantly increased by ethanol extract of PHS at 0.25, 0.50, and 1.00 mg/mL (Fig. 1a). Prolonged exposure to 0.25, 0.50 and 1.00 mg/mL of PHS significantly reduced the lifespan of *C. elegans* (Fig. 1b).

### Effects of PHS exposure on development and reproduction of *C. elegans*

The evaluation indexes of development of *C. elegans* include three aspects: body length, body width and abnormal development. Changes of body length reflect the growth rate and physiological state of *C. elegans*. In this study, we chose body length as a development evaluation indicator. Prolonged exposure to 0.25 mg/mL of PHS did not obviously influence the body length of *C. elegans*, but prolonged exposure to 0.50, 1.00 mg/mL of PHS significantly reduced the body length of *C. elegans* (Fig. 1c)*.*

The evaluation indexes of reproduction of *C. elegans* include three aspects: generation time, brood size and number of eggs in the uterus. The generation time is used primarily to investigate the reproductive speed of *C. elegans*. Brood size is mainly used to investigate the reproductive ability of *C. elegans*, and the number of eggs in the uterus can simultaneously examine the reproductive capacity and the physiological function of the ovipositor. In this study, we chose brood size as a reproduction evaluation indicator. We observed that prolonged exposure to 0.25, 0.50 mg/mL of PHS did not significantly influence the brood size of *C. elegans*, but prolonged exposure to 1.00 mg/mL of PHS significantly reduced the brood size of *C. elegans* (Fig. 1d)*.*

### Effects of PHS exposure on locomotion behavior of *C. elegans*

Evaluation indexes of locomotion behavior of *C. elegans* include four aspects: head thrash, body bend, basic movement and locomotion rate. Locomotion behavior, as an index reflecting the basic function of the nervous system, has been widely used in the analysis of neurotoxicity. In this study, we chose head thrash and body bend as locomotion behaviors evaluation indicator. Prolonged exposure to all the examined concentrations of PHS significantly decreased head thrash of *C. elegans* (Fig. 1e). Prolonged exposure to 0.25 mg/mL of PHS did not significantly influence the body bend of *C. elegans*, but prolonged exposure to 0.50, 1.00 g/mL of PHS significantly decreased the body bend of *C. elegans* (Fig. 1f)*.*

### Effects of PHS exposure on AChE activity of *C. elegans*

**Fig. 2:**
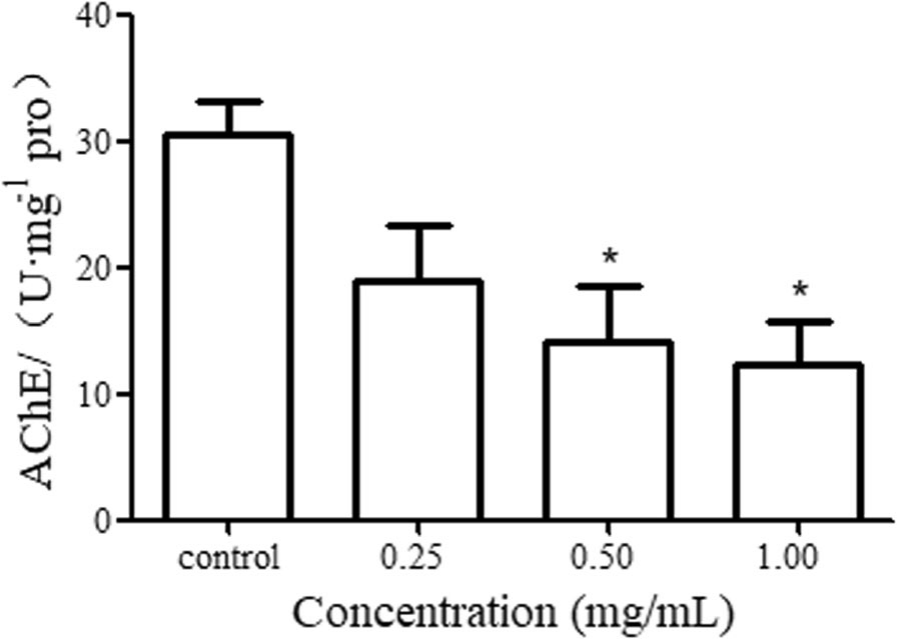
Effects of prolonged exposure to PHS on AChE activity of *C. elegans.* Exposures were performed from L4 stage to young adult. PHS, seeds of *Peganum harmala* L.. *C.elegans* L4 stage to young adults were exposed to PHS. *P < 0.05

*C. elegans* is a model animal with simple central nervous system, which is similar to vertebrates in neurophysiology. *C. elegans* can be used to estimate the harmful effects of toxic substances on the biological nervous system. AChE activity is a classic indicator of neurotoxicity. AChE plays an important role in the process of biological nerve conduction. In cholinergic synapses, AChE can degrade acetylcholine and stop the excitatory effect of neurotransmitters on the post-synaptic membrane, thus ensuring the normal transmission of nerve signals in the body. Prolonged exposure to 0.25 mg/mL of PHS did not significantly influence the AChE activity of *C. elegans*. But prolonged exposure to 0.50, 1.00 mg/mL of PHS significantly decreased the AChE activity of *C. elegans* (Fig. 2). This result implied that prolonged exposure to PHS may damage the nervous system of *C. elegans*.

### Heat stress resistance property of PHS in *C. elegans*

Heat stress is the sum of non-specific responses produced by an animal when it is stimulated by high temperatures that exceed its body’s ability to regulate temperature. Too high temperature disrupts the normal heat balance of the organism, and the body will produce a series of complex physiological and psychological changes to deal with this change. Heat stress is a commonly used experiment in aging research. To investigate whether PHS has a stress resistance property, *C. elegans* pretreated with 0.25, 0.50, and 1.00 mg/mL of PHS ethanol extract for 24 h were further exposed to 35 °C.

**Fig. 3:**
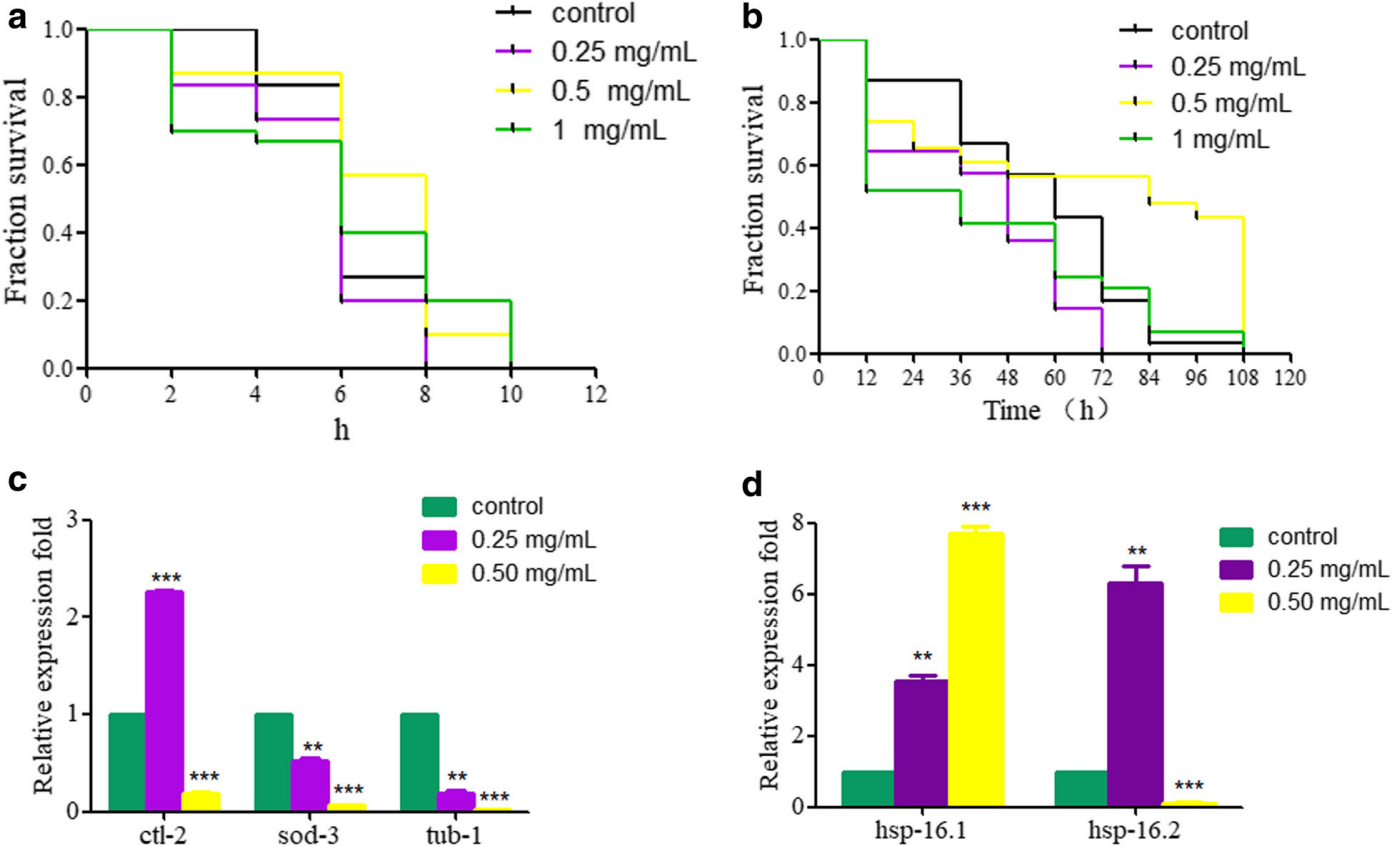
**a** Heat-stress resistance of PHS-treated *C. elegans* **b** Oxidative stress resistance of PHS-treated *C. elegans* **c**, **d** Expression patterns of genes required for development in control and PHS exposed nematodes. The results were expressed as the relative expression ratio between the targeted gene and the reference *GAPDH* gene. Exposures were performed from L4-larvae to young adult. PHS, seeds of *Peganum harmala* L.. *C.elegans* L4-larvae to young adults were exposed to PHS. **P < 0.01, ***P < 0.001

Compared with the control group, the lifespan of PHS with different concentrations was significantly longer than that of the control group, which indicated that PHS could significantly improve the heat resistance of *C. elegans* (Fig. 3a).

### Oxidative stress resistance property of PHS in *C. elegans*

Oxidative stress is a negative effect of free radicals in the body and is considered to be an important factor leading to aging and disease. Paraquat is a strong oxidant commonly used in oxidative stress experiments. After paraquat enters the *C. elegans*, a series of biochemical reactions produce superoxide anions that are harmful to the cells, and the superoxide anions are further converted into active oxygen clusters such as hydrogen peroxide and hydroxyl radicals [24].

In this experiment, each experimental group was treated with 50 mM paraquat. Compared with the control group, PHS significantly prolonged the lifespan of *C. elegans*, indicating that PHS has certain antioxidant capacity (Fig. 3b).

### Effect of PHS on related gene expressions in *C. elegans*

Gene expression is an effective indicator for evaluating the effects of foreign compounds, and the specific toxic effects of foreign compounds can be determined according to the characteristics of target genes. *Hsp-l6.l* and *hsp-l6.2* are heat shock protein genes. *Sod-3* is the superoxide dismutase gene. *Ctl-2* is the catalase gene. *Tub-1* is a gene related to longevity regulation.

After exposed to 0.25 and 0.50 mg/mL of PHS ethanol extract for 24 h, we observed that 0.25 mg/mL of PHS significantly increased the expression levels of *ctl-2*, *hsp-16.1* and *hsp-16.2* genes, and decreased the expression levels of *sod-3* and *tub-1*. And 0.50 mg/mL of PHS significantly increased the expression levels of *hsp-16.1* gene, and decreased the expression levels of *hsp-16.2*, *ctl-2*, *sod-3* and *tub-1*genes (Fig. 3c, d).

### Determination of IC_50_ values

The DPPH method is a commonly used method to evaluate and screen the antioxidant activity of substances in vitro. It has the advantages of simple operation, high sensitivity and good reproducibility. IC_50_ is the antioxidant concentration when the DPPH concentration drops to 50%. The lower the IC_50_, the stronger the free radical scavenging ability of the antioxidant; the higher the IC_50_, the weaker the scavenging activity.

**Fig. 4:**
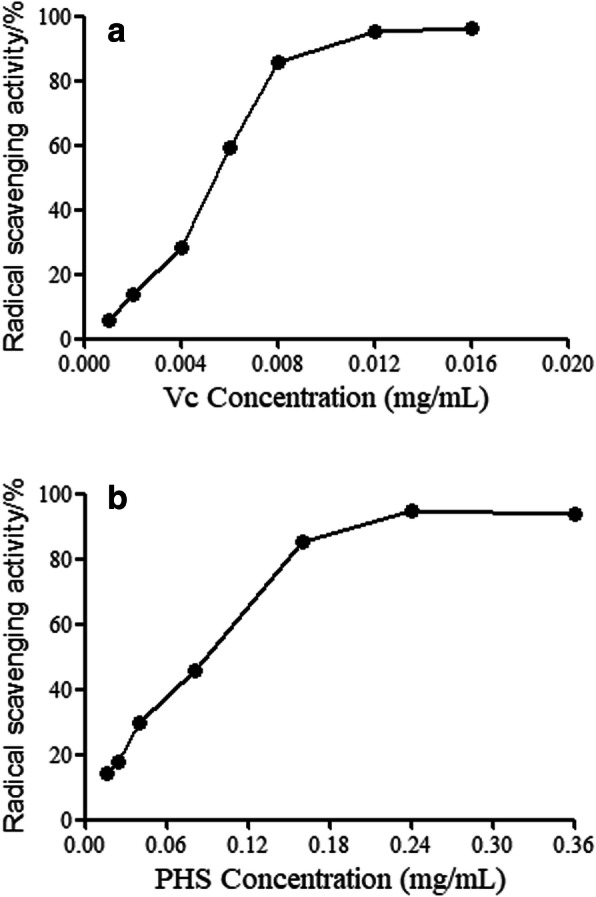
**a** The radical scavenging activity of Vc **b** The radical scavenging activity of PHS

The 50% radical scavenging activity (IC_50_) of Vc is 0.005 mg/mL (Fig. 4a). The 50% radical scavenging activity (IC_50_) of PHS is 0.087 mg/mL (Fig. 4b).

### Identification of the main chemical components in raw-processed PHS

The content of alkaloids in PHS is 4 to 6%, which is mainly alkaloids such as harmaline, harmine. Harmaline and harmine are not only the main chemical constituents but also the main active constituents.

**Fig. 5:**
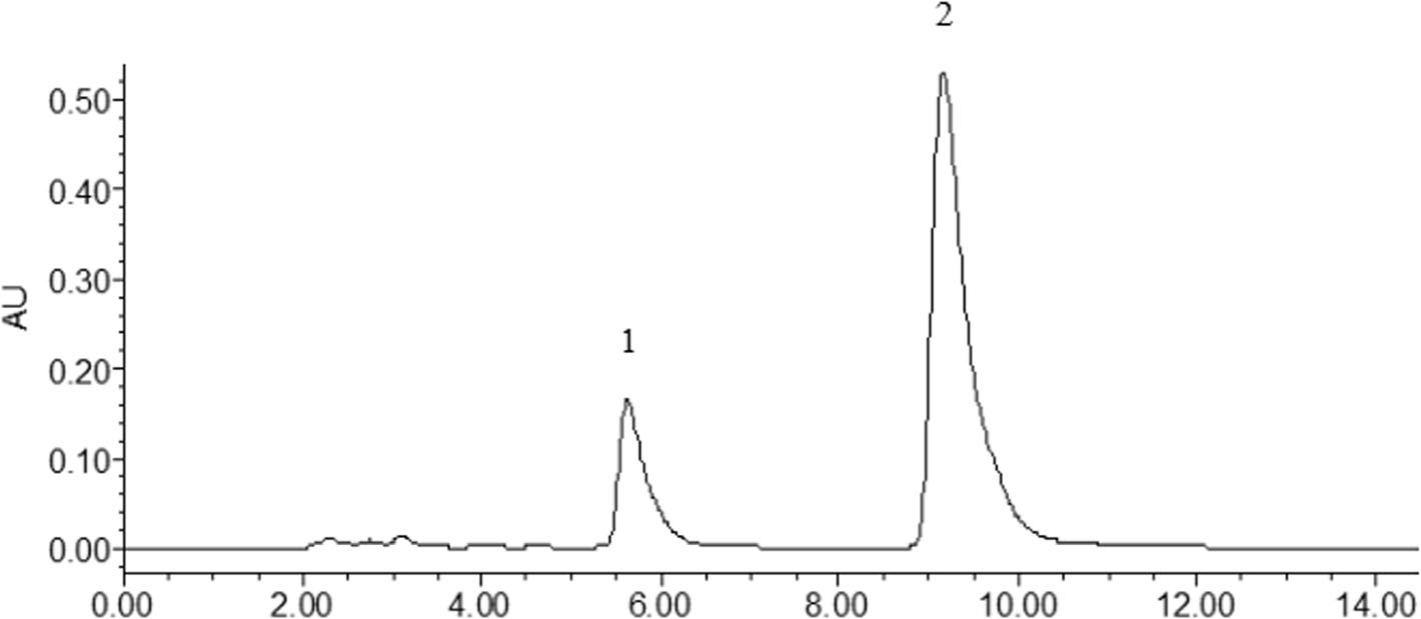
Chemical components in PHS analyzed by HPLC.1, harmaline; 2, harmine

Using the HPLC, we identified the main chemical components in the concentrated PHS solution (Fig. 5a). The content of harmaline was 96.76 mg/g and the content of harmine was 98.05 mg/g.

## Discussion

PHS has been used as a folk medicine for a long time in both oriental and western counties [25]. In this study, we conducted a comprehensive assessment of PHS toxicity using *C. elegans*. We evaluated the PHS toxicity of L4 stage for prolonged exposure. This period was chosed to coincide with the developmental stage when the number of germ cells reached its maximum, but before the embryonic membrane became impermeable [26].

We proposed a hypothesis to explain the underlying mechanism of PHS toxicity: the toxicity of PHS may be attributed to its combinational effects on nervous system and Insulin/IGF-1 signaling pathway. Our data showed that prolonged exposure to PHS could be toxic to the lifespan (Fig. 1a, b), development (Fig. 1c), reproduction (Fig. 1d) and locomotion behavior (Fig. 1e, f) of *C. elegans*. This may be closely related to the neurotoxicity of PHS [9]. The changes in the behavior of *C. elegans* are mainly controlled by a variety of neurons and their mutual nerve conduction [27]. Acetylcholine is closely related to many behaviors of *C. elegans*, and locomotion behavior is the most important acetylcholine-related behavior [28]. Metrifonate and haloxon can lead to the loss of kinetism by inhibiting the activity of AChE in *C. elegans* [29]. Harmaline and harmol showed good inhibitory activities against AChE [30]. The AChE activity of control *C. elegans* was 30.61 U/mg pro and exposure to 1.00 mg/mL of PHS was 12.36 U/mg pro. Our data showed PHS could obviously decrease the AChE activity of *C. elegans*.

In *C. elegans*, *daf-2*, *tub-1*, *ctl-2*, *hsp-16.1*, *hsp-16.2* are required for Insulin/IGF-1 signaling pathway [31]. In *C. elegans*, this pathway plays a key role in the regulation of development, metabolism and aging, and is the most detailed life-regulating pathway in current research [32]. We found the expression change of these genes (Fig. 3c, d), it may be related to the reduced lifespan of *C. elegans.*

Previous studies have indicated PHS possess certain antioxidant activity. Beta-carbolines (harmaline and harmine) may prevent dopamine-induced mitochondrial damage and PC12 cell death through a scavenging action on reactive oxygen species and inhibition of monoamine oxidase and thiol oxidation [5]. The beta-carboline alkaloids have a significant protective effect against H_2_O_2_ and paraquat oxidative agents in yeast cells, that their ability to scavenge hydroxyl radicals contributes to their antimutagenic and antigenotoxic effects [33]. Aqueous extract of *Peganum harmala* could prevent symptoms and reduced oxidative stress markers in rats with Parkinson’s disease induced by 6-hydroxydopamine [34]. Our data also suggest that pretreatment with PHS induced resistance properties in *C. elegans* against damage from both heat-stress (Fig. 3a) and oxidative stress (Fig. 3d). Our data further support the protective effects of PHS on animals. The mechanism to protects against heat resistance needs further study.

Our study indicates the potential adverse effects of PHS in *C. elegans* after long-term exposure. These results lay the foundation for further study of the pharmacological mechanism of PHS, and contribute to the reasonable application of PHS on clinical practice. At least according to our data, the duration and dose should be carefully considered before using the drug. At the same time, it is also possible to design and consider strategies such as modulation of the process to reduce the possible adverse effects of PHS.

In summary, with the PHS as a sample, our study provide evidence to illustrate the application value of the system in the evaluation of the toxicity of Chinese medicinal materials. After long-term exposure, PHS had a variety of toxic effects in *C. elegans*, which may be the result of a combination of the nervous system and Insulin/IGF-1 signaling pathway. In the future, the detection of the beneficial or adverse effects of specific chemical constituents in PHS will help us further understand and control the toxicity formation and clinical use of this drug.

Previous studies have shown that *C. elegans* can be used for toxicity assessment of metal [35], organic contaminants [36], pharmaceutical compounds [37] or specific components extracted from plants [38]. In the present study, our data further suggests that *C. elegans* can be utilized to toxicity assessment of Chinese medicinal materials. Therefore, *C. elegans* can be used to estimate the beneficial effects and adverse reactions of Chinese medicinal materials. We first evaluated the toxicity of PHS using *C. elegans*. Since most Chinese medicinal materials still lack detailed toxicity data, *C. elegans* will be a fast and systematic system for assessing the toxicity of Chinese medicinal materials.

However, *C. elegans* is largely different from the human species and lack organs such as heart, kidney, lung, etc. [39] Therefore *C. elegans* cannot completely replace traditional animal toxicology experiments such as rats. In the prepared PHS solution, there may be various chemical components. We only studied the toxicity of ethanol extract on *C. elegans* which means that our data may not fully reflect the biological effects of PHS prepared by other extraction methods. The toxicological effects of PHS and related toxicological mechanisms are rarely documented. For these reasons, extensive pharmacological and chemical experiments, as well as human metabolism, should be the focus of future research.

## Conclusions

Our study fully evaluated the toxicity effects of PHS on *C. elegans*, investigated the possible mechanism of the toxicity effects of PHS, and provided pharmacological research paradigm for the study of traditional medicine toxicity. The results will be helpful in understanding the probable toxicity effects of PHS and its underlying mechanism. In addition, the results provided new ideas and methods for the biological evaluation of the toxicity of Chinese medicinal materials.

## Data Availability

The datasets used and/or analyzed during the current study are available from the corresponding author upon reasonable request.

## Abbreviations

AChE: Acetylcholinesterase
*C. elegans*: *Caenorhabditis elegans*
*E. coli* OP50: *Escherichia coli* OP50
HPLC: High performance liquid chromatography
IC_50_: Half-inhibitory concentration values
NGM: Nematode growth medium
PHS: *Peganum harmala* L. seeds
qRT-PCR: Quantitative real-time polymerase chain reaction
TCM: Traditional Chinese medicine
Vc: Vitamin C
